# Healthcare Utilization and Recovery Duration After Ankle Fracture: A Claims‐Based Study of Timeline, Costs and Complications

**DOI:** 10.1111/jep.70450

**Published:** 2026-05-03

**Authors:** Robert B. Weinstein DPM, Samantha J. Beckley, Maha Karim, Shaun K. Stinton, Thomas P. Branch

**Affiliations:** ^1^ Ankle and Foot Centers of Georgia Atlanta Georgia USA; ^2^ ArthroResearch LLC Atlanta Georgia USA; ^3^ Ermi LLC Atlanta Georgia USA

**Keywords:** ankle fracture, complications, contracture, healthcare claims, healthcare costs, recovery duration, rehabilitation

## Abstract

**Rationale:**

Ankle fractures are common orthopaedic injuries with wide variability in recovery duration and outcome. While long‐term outcomes are well documented, less is known about the short‐term recovery period. An updated analysis using claims data would provide greater clarity on short‐term recovery patterns.

**Aims and Objectives:**

The study aimed to characterise recovery duration after treatment for ankle fracture and to evaluate associations between comorbidities, perioperative complications, additional procedures and healthcare costs.

**Method:**

Healthcare claims from the IBM MarketScan database (2015–2018) were analysed to determine recovery costs for the index treatment and subsequent events including revision surgery, motion restoring surgery (MRS), rehospitalizations, and complication‐related interventions. Recovery duration was defined as the interval between the initial surgery/treatment and the final physical/occupational therapy claim. Outcomes were summarised using medians and interquartile ranges (IQR).

**Results:**

Among 7,112 patients, the median index treatment cost was $5163 (IQR: $994–$12,444), and the recovery duration was 88 days (IQR: 36–492). Thirty‐eight percent of patients required more than 6 months to complete recovery. Post‐treatment complications were associated with markedly longer and more expensive recovery. Patients who required a complication‐related surgery had a recovery duration that was 4 times longer and incurred costs that were 8 times greater than those without such events. Joint contracture and MRS were strongly associated with prolonged and costly recoveries.

**Conclusion:**

This claims‑based analysis identified wide variation in short‑term recovery after ankle fracture. Strong associations were demonstrated between complications, including joint contracture, MRS and rehospitalizations, and extended recovery duration and higher costs. These findings may help clinicians identify patients at risk for delayed recovery and support more informed decision‑making in early post‑treatment care.

## 1 Introduction

Ankle fractures are among the most common lower‐extremity injuries, with a reported incidence of 4.22 per 10,000 person‐years equating to approximately 141,000 cases annually in the United States [1]. These injuries often lead to prolonged pain, functional limitation at home and at work, and reduced quality of life [2, 3]. While many ankle fractures can be managed effectively with early mobilization, more complex injuries requiring surgical fixation or extended immobilization are prone to delayed functional recovery, postoperative stiffness and ankle or Achilles tendon contracture [4, 5]. Comorbidities such as diabetes, peripheral vascular disease (PVD) and cardiovascular disease (CVD) can further slow recovery, increase complication risk and drive higher costs [6, 7, 8]. Together, these factors can contribute to substantial variability in reported outcomes after ankle fracture and overall healthcare utilization.

Most previous studies have focused on long‐term outcomes, typically 2 plus years post‐injury. While many patients regain acceptable function, persistent symptoms were common and lead to long‐term functional impairment in 25%–40% of patients [9, 10, 11]. However, short‐term recovery outcomes, which include a critical period of recovery, complication management, and return to function, remain comparatively understudied. This early recovery phase is also the period of highest healthcare utilization and cost accumulation, making it highly relevant for clinical decision‐making, patient counselling and resource allocation [12, 13]. A clearer understanding of this short‐term recovery trajectory could help identify patients at risk for delayed recovery and inform strategies to improve outcomes and reduce utilization.

To address this evidence gap, we used the IBM MarketScan Commercial Claims and Encounters Database, a comprehensive national dataset containing real‐world healthcare utilization, cost and outcomes data for musculoskeletal injury across insured populations [14]. Its large sample size and detailed information allow for characterization of recovery patterns including clinically meaningful rare events such as motion restoring surgery (MRS) or rehospitalization, which are difficult to capture in smaller prospective studies. While there are limitations with claims‐based research such as a lack of clinical detail such as fracture severity, functional scores and radiography, claims data provide an excellent view of real‐world recovery patterns and associated costs on a large scale.

The primary objective of this study was to define a benchmark of short‐term recovery following ankle fracture, defined as the period from initial treatment through completion of physical or occupational therapy, using healthcare claims data. Secondary objectives were to evaluate how comorbidities and post‐treatment complications including joint contracture, rehospitalizations and additional procedures affect recovery duration and total healthcare costs.

## 2 Methods

### 2.1 Study Population

This retrospective study analysed healthcare claims data from the IBM MarketScan Commercial Claims and Encounters Database (2015–2018). The database included detailed, longitudinal, de‐identified records of inpatient and outpatient encounters, procedures and payments for patients that were covered by employer‐sponsored or commercial insurance.

Inclusion criteria were:

1. A primary ankle fracture identified by Current Procedural Terminology (CPT) codes for open or closed treatment (Table S1).
2. Continuous coverage under a single insurance carrier for at least 2 consecutive years, as defined by the IBM MarketScan database.
3. At least one post‐treatment physical therapy (PT) or occupational therapy (OT) CPT charge (codes ≥ 97,000 and < 98,000) to confirm participation in rehabilitation.
4. Age limits were defined by the IBM MarketScan database to include all covered individuals (all ages).

Exclusion criteria included individuals with bilateral ankle fracture (either simultaneous or staged) to prevent bias in recovery duration and cost estimates.

This study followed the Declaration of Helsinki's ethical guidelines and was deemed exempt from Institutional Review Board review due to the use of de‐identified data. As a result, consent to participate did not apply.

### 2.2 Cost Analysis

All reported costs were in U.S. dollars and correspond to the same time period as the MarketScan data (2015–2018). Costs associated with the index surgery/treatment represent all healthcare dollars spent during inpatient hospitalization or within 8 days of the outpatient treatment. Total Costs represent all healthcare dollars spent which included the cited index treatment and any additional post‐index treatment events. Therefore, these costs are a true representation of the event and the risk of related subsequent events. Standard CPT codes for post‐treatment costs, such as PT/OT (≥ 97,000 and < 98,000), physician visits (≥ 99,200 and < 99,300, plus injection CPT codes 20610/20611), and radiology, were also analysed (see Table S2). Costs unrelated to ankle fracture were excluded from the analysis by absence of ankle‐related International Classification of Disease (ICD) 9/10 codes on the Healthcare Common Procedure Coding System (HCPCS) form. All reported costs reflect insurance‐paid amounts.

### 2.3 Index Surgery/Treatment Costs

All expenses related to the initial surgery or treatment were included, identified by the CaseID for inpatient surgery. For surgeries or treatments performed in outpatient settings where no CaseID was available, all costs incurred within an 8‐day window were included in the total index costs to account for claims submitted over time. In cases where an ankle fracture procedure began in an outpatient setting but the patient was transferred to a hospital within 8 days, these costs were included in the outpatient index surgery expenses. Patients undergoing inpatient surgery followed by a transfer to an inpatient rehabilitation facility were treated as having two distinct hospitalizations, with the rehabilitation stay classified as a nonoperative hospitalization. The distribution of open versus closed treatment of ankle fractures is in Table 1 with 56% receiving closed treatment.

**Table 1:** Frequency of open and closed treatment of ankle fractures.

Closed treatment of ankle fractures	Count
Closed treatment of ankle dislocation; requiring anaesthesia, with or without percutaneous skeletal	36
Closed treatment of ankle dislocation; without anaesthesia	170
Closed treatment of bimalleolar ankle fracture, (including Potts); with manipulation	261
Closed treatment of bimalleolar ankle fracture, (including Potts); without manipulation	185
Closed treatment of distal fibular fracture (lateral malleolus); with manipulation	189
Closed treatment of distal fibular fracture (lateral malleolus); without manipulation	2002
Closed treatment of fracture of weight bearing articular portion of distal tibia (e.g. pilon or tibial plafond), with or without anaesthesia; with skeletal traction and/or requiring manipulation	80
Closed treatment of fracture of weight bearing articular portion of distal tibia (e.g. pilon or tibial plafond), with or without anaesthesia; without manipulation	112
Closed treatment of medial malleolus fracture; with manipulation, with or without skin or skeletal	49
Closed treatment of medial malleolus fracture; without manipulation	269
Closed treatment of proximal fibula or shaft fracture; with manipulation	37
Closed treatment of proximal fibula or shaft fracture; without manipulation	235
Closed treatment of proximal tibiofibular joint dislocation; without anaesthesia	1
Closed treatment of trimalleolar ankle fracture; with manipulation	287
Closed treatment of trimalleolar ankle fracture; without manipulation	42
**Total**	**3955**

**Open treatment of ankle fractures**	**Count**
Open treatment of ankle dislocation, with or without percutaneous skeletal fixation; with repair or	12
Open treatment of ankle dislocation, with or without percutaneous skeletal fixation; without repair or	1
Open treatment of bimalleolar ankle fracture, with or without internal or external fixation	735
Open treatment of distal fibular fracture (lateral malleolus), with or without internal or external	1127
Open treatment of distal tibiofibular joint (syndesmosis) disruption, with or without internal or	219
Open treatment of fracture of weight bearing articular surface/portion of distal tibia (e.g. pilon or tibial plafond), with internal or external fixation; of both tibia and fibula	114
Open treatment of fracture of weight bearing articular surface/portion of distal tibia (e.g. pilon or tibial plafond), with internal or external fixation; of fibula only	14
Open treatment of fracture of weight bearing articular surface/portion of distal tibia (e.g. pilon or tibial plafond), with internal or external fixation; of tibia only	193
Open treatment of medial malleolus fracture, with or without internal or external fixation	227
Open treatment of proximal fibula or shaft fracture, with or without internal or external fixation	77
Open treatment of proximal tibiofibular joint dislocation, with or without internal or external fixation, or with excision of proximal fibula	2
Open treatment of trimalleolar ankle fracture, with or without internal or external fixation, medial and/or lateral malleolus; with fixation of posterior lip	87
Open treatment of trimalleolar ankle fracture, with or without internal or external fixation, medial and/or lateral malleolus; without fixation of posterior lip	349
**Total**	**3157**

### 2.4 Recovery Period Duration

The post‐treatment recovery period was defined as the time span from the index ankle fracture treatment (surgery or casting) to the date of the final PT or OT claim. Because this study was limited to claims data, the last PT/OT claim was used as a proxy for the end of treatment. Without the availability of detailed individual medical records, it was not possible to independently validate the true end of therapy.

### 2.5 Secondary Interventions and Rehospitalizations

The effects of post‐treatment complications on recovery were evaluated, with particular attention to rehospitalizations both with and without additional procedures. This included ankle fracture revisions (Table S3), motion restoring surgeries (MRS) (Table S4) and surgeries addressing other complications (Table S5). Rehospitalizations were identified through a new CaseID associated with or occurring after the initial treatment.

### 2.6 Ankle Joint Contracture

The effect of either ankle or Achilles tendon contracture at the time of index surgery/treatment or after the index surgery/treatment was also analysed (Table S6).

### 2.7 Comorbidities

This study evaluated the impact of comorbidities on recovery timelines by analysing specific patient subsets. Groups were identified based on the presence of diabetes (ICD‐9/10 codes 25.0, E11.9), obesity (codes 278., E66., Z68.4), PVD (codes 440.:444., 785., I73.9), joint infections (codes 711., 996., M00., M01, M02), cardiovascular disease (CVD; codes 390.:459., I11, I20, I21, I25) and ankle contracture (codes 718.47, M24.57). The objective of this study was not to develop or compare composite comorbidity indices such as the Charlson or Elixhauser indices but rather to assess the effect of individual conditions on post‐treatment recovery. Future work using these indices could provide a more comprehensive analysis of comorbidity burden. Post‐treatment complications including the incidence rates of infection (Table S7) and pulmonary embolism (Table S8) were also analysed.

### 2.8 Data Analysis

Data were analysed using the R statistical programming language (version 4.3.3). Due to the large sample size and non‐normal distribution, results are reported as medians along with the IQR, representing the 25th to 75th percentiles. Mood's Median Tests were used for group comparisons as the dataset was large and not suitable for means‐based tests. No confidence intervals were computed given the descriptive focus of the study.

### 2.9 Presentation of Recovery Pathway

**Figure 1:**
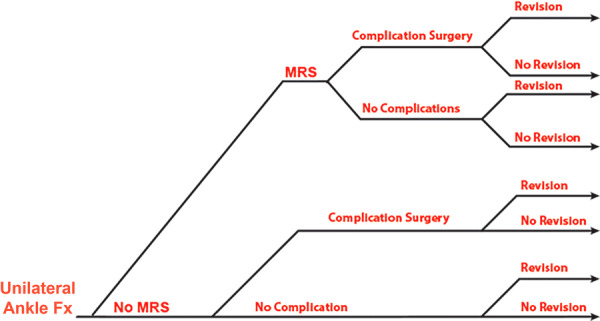
Schematic diagram representing the potential recovery pathways for unilateral ankle fracture patients. This does not represent a specific timeline. MRS, motion restoring surgery.

An uncomplicated recovery pathway would be a course in which the patient undergoes the initial treatment, is discharged, participates in PT, and achieves recovery within 6 months. It is well established that injuries or surgeries transition from acute to chronic after the 6‐month mark, and insurance providers limit temporary or short‐term disability coverage to this timeframe [15, 16]. To illustrate the complexities of recovery, a multi‐pathway chart (Figure 1) demonstrates the combined impact of various post‐treatment events, with a particular focus on the increased risks associated with MRS decisions. Rather than depicting a specific timeline, the chart emphasises the cumulative risk of successive complications during the recovery period.

## 3 Results

### 3.1 Study Population

**Figure 2:**
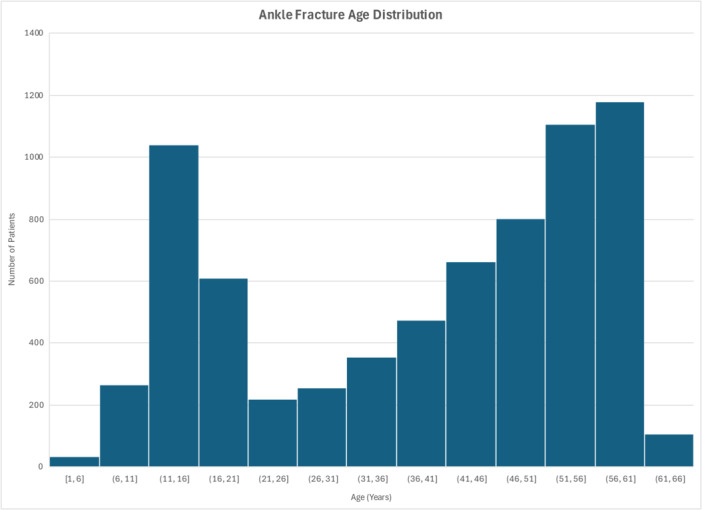
Age distribution of 7112 unilateral ankle fracture patients included in the study.

A total of 7112 ankle fracture patients within the IBM Watson Database had a minimum of 2 years continuous coverage. The group was 60.6% female and 39.4% male. The median age was 44 years with a range from 1 to 62 years (IQR: 20–54 years) (Figure 2). Height and weight data were not available.

### 3.2 Index Surgery/Treatment Costs

Of the 7112 patients, 596 were treated as an inpatient only, 6397 were outpatient and 119 started as outpatient, but ended as inpatient. The median cost of the index surgery/treatment was $5163 (IQR: $994–$12,444). The median inpatient surgery/treatment cost was $14,567 (IQR: $3223–$27,390). The median cost of outpatient treatment was $4683 (IQR: $893–$10,984). If the patient started as an outpatient but ended as an inpatient the median cost was $23,788 (IQR: $17,658–$31,921).

### 3.3 Recovery Period Duration

**Figure 3:**
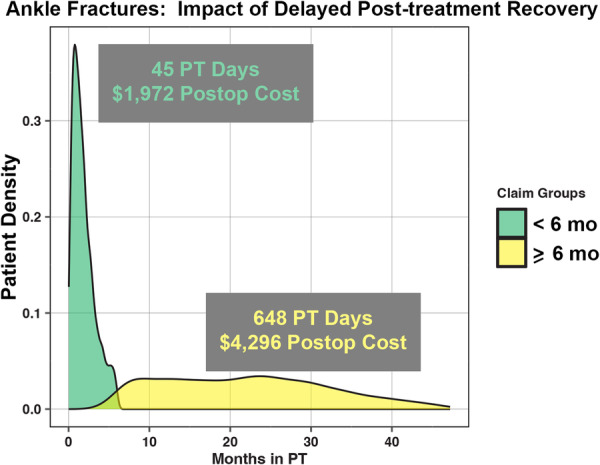
Distribution of recovery time for unilateral ankle fracture patients. Graph depicting the bimodal distribution of the days spent in physical therapy in patients that completed their treatment within (green) or longer than (yellow) 6 months.

The median length of post‐treatment recovery (time between index surgery/treatment and last PT claim) for a unilateral ankle fracture was 88 days (IQR: 36–492 days). Only 62% of patients completed their post‐treatment period in 6 months with 38% of patients taking over 6 months to complete PT (Figure 3). Patients who completed their post‐treatment course in less than 6 months, spent a median of 45 days in structured outpatient PT whereas patients who completed their post‐treatment course in over 6 months, spent a median of 648 days in structured outpatient PT. The additional post‐treatment costs incurred after 6 months for patients undergoing PT beyond that point were a median of $2324 (IQR: $1029–$4885).

### 3.4 Secondary Interventions and Rehospitalizations

We examined whether a difference exists in the length of the post‐treatment recovery period after four major events that may occur after a unilateral ankle fracture: (1) Revision surgery after ankle fracture; (2) MRS; (3) Complication surgery related to the ankle fracture and (4) Nonoperative Hospitalizations related to infection or pulmonary embolus in the post‐treatment period.

**Figure 4:**
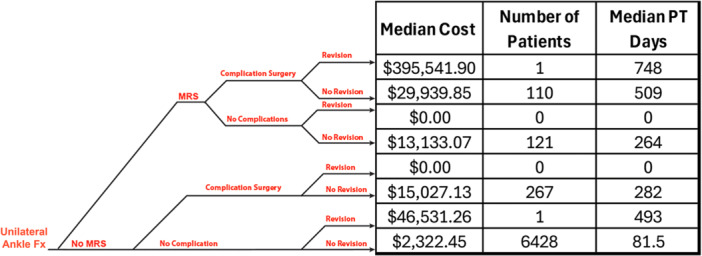
The cumulative impact of multiple events on recovery costs and time to recover. A schematic diagram illustrating potential recovery pathways for patients with a unilateral ankle fracture. The diagram includes the percentage of patients in each scenario, median costs and recovery time (measured in physical therapy days). This representation is not to scale and does not depict a specific timeline. MRS, motion‐restoring surgery; PT, physical therapy.

Patients with ankle revision surgery (salvage) took 6 times longer and cost 31 times those patients not requiring revision; however the risk of revision is quite low (Table 2). After MRS, such as a manipulation under anaesthesia (MUA), arthroscopy for lysis of adhesions or synovectomy, the median number of days in structured PT and the costs of post‐treatment care both nearly tripled (Table 2). The median recovery period and cost for patients that required a complication surgery after a unilateral ankle fracture were 4 times longer and added 8 times the cost in comparison to patients that did not require any complication surgeries (Table 2). Nonoperative hospitalizations for pulmonary embolus and/or infection increased the post‐treatment period nearly 2 times (191 vs. 86 days) at a median cost of $30,025 per event (Table 2). The cumulative effect on medical care costs and recovery time as a result of multiple events in the post‐treatment period can be seen in Figure 4 and Table 3. In particular, the decision to perform a MRS substantially impacted the risk of requiring a complication surgery, with patients undergoing MRS being 12 times more likely to need complication surgery compared to those without a MRS (47% vs. 4%).

**Table 2:** Treatment costs and recovery times with and without the major events: (1) Motion Restoring Surgery (MRS), (2) Complication Surgery, (3) Ankle Fracture Revision Surgery and (4) Nonoperative Hospitalization.

	Major events			
	MRS	Complication surgery	Revision surgery	Non‐operative hospitalization
**Cost without event**	2443	2393	2541	2481
**Interquartile range**	(1305–47,43)	(1292–4,552)	(1332–5094)	(1318–4882)
**Cost with event**	19,698	19,961	78,206	30,025
**Interquartile range**	(11,477–33,997)	(9848–36,961)	(44,364–198,208)	(17,571–59,859)
**Cost comparison *p*‐value**	< 0.01	< 0.01	0.07*	< 0.01
**PT days without**	85	84	88	86
**Interquartile range**	(36–469)	(35–462)	(36–491)	(36–484)
**PT days with**	357	330	493	191
**Interquartile range**	(85–750)	(71–721)	(37–551)	(63–710)
**PT days comparison *p*‐value**	< 0.01	< 0.01	0.99*	< 0.01
**Number of patients without event (*n*)**	6880	6705	7107	6975
**Number of patients with event (*n*)**	232	407	5	137
**Infection without event (%)**	2.7	2.4	2.8	2.6
**Infection with event (%)**	9.1	10.3	80.0	19.7
**PE without event (%)**	3.6	3.4	3.6	3.4
**PE with event (%)**	3.9	6.4	20.0	12.4

*Note:* Mood's Median tests were used for comparisons.

Costs and days in physical therapy (PT) are presented as medians and interquartile ranges.

Abbreviation: PE, pulmonary embolism.

* *p*‐Values are not significant due to the very small number of patients who underwent revision surgery and the high variability in those patients.

**Table 3:** Cumulative effect of multiple events in the unilateral ankle fracture patients postoperative period.

Event sets	*N*	Patients (%)	Cost ($)	IQR	PT (days)	IQR
**MRS + Complication Surgery/OPS + Rev**	1	0.0	395,542	N/A	748	N/A
**MRS + Complication Surgery/OPS** + **No Rev**	110	1.5	29,940	(11,113–101,836)	509	(21–1203)
**MRS + Rev** + **No Complication Surgery/OPS**	0	0.0	0	N/A	0	N/A
**MRS** + **No Rev** + **No Complication Surgery/OPS**	121	1.7	13,133	(4546–43,650)	264	(17–1133)
**No MRS + Complication Surgery/OPS + Rev**	0	0.0	0	N/A	0	N/A
**No MRS + Complication Surgery/OPS** + **No Rev**	267	3.8	15,027	(1686–99,405)	282	(14–1083)
**No MRS + Rev** + **No Complication Surgery/OPS**	1	0.0	46,531	N/A	493	(493–493)
**No MRS** + **No Rev** + **No Complication Surgery/OPS**	6428	90.4	2322	(487–11,015)	81.5	(11–1003)

*Note:* Costs and days in physical therapy (PT) are presented with medians and interquartile ranges (IQR).

Abbreviations: MRS, motion restoring surgery; OPS, outpatient surgery; Rev, revision.

A single ankle fracture with no complications, no revisions and no MRS had a median post‐index treatment cost of $2322 (Figure 4). We found that 7% of single ankle fractures required a secondary surgery, including revisions, MRS and complication surgeries (Figure 4). This median cost surged dramatically to $78,205 with a revision surgery (Table 2). Out of the 7112 patients that were observed with a unilateral ankle fracture, 6880 patients did not undergo a MRS. These patients had a median of 85 PT days and the median post‐treatment cost was $2443 (Table 2). The median cost for patients that had MRS was $19,698 (IQR: $11,477–$33,997) with a median of 357 PT days (Table 2).

### 3.5 Ankle Joint Contracture

The presence of either ankle or Achilles tendon contracture at the time of index surgery/treatment or after the index surgery/treatment doubled the post‐treatment cost compared to patients without contracture. The median recovery time for both patients who had a contracture pre‐treatment and those that had a contracture post‐treatment were significantly greater than in the subset of patients who did not develop a contracture (6 times greater and 4 times greater, respectively, Table 4).

**Table 4:** The impact of the diagnosis of joint contracture on the costs and recovery time in the unilateral ankle fracture postoperative period.

	Joint contracture	
	Before treatment	After treatment
**Cost without event**	2542	2539
**Interquartile range**	(1333–5105)	(1331–5093)
**Cost with event**	5068	4997
**Interquartile range**	(443–46,531)	(2782–29,361)
**Cost comparison *p*‐value**	0.68*	< 0.01
**PT days without event**	88	87
**Interquartile range**	(36–491)	(36–490)
**PT days with event**	537	352
**Interquartile range**	(63–754)	(57–685)
**PT days comparison *p*‐value**	0.22*	0.05
**Number of patients without event (*n*)**	7106	7090
**Number of patients with event (*n*)**	6	22
**Infection without event (%)**	2.8	2.8
**Infection with event (%)**	50.0	18.2
**PE without event (%)**	3.6	3.6
**PE with event (%)**	0.0	9.1

*Note:* Mood's Median tests were used for comparisons.

Costs and days in physical therapy (PT) are presented as medians and interquartile ranges.

Abbreviation: PE, pulmonary embolism.

* *p*‐Values are not significant due to the very small number of patients who had joint contracture at the time of surgery and the high variability in those patients.

### 3.6 Comorbidities

The effect of the presence of diabetes, obesity, PVD and CVD is shown in Table 5. The clinical impact of these conditions on recovery was small, however, there were a larger number of pulmonary embolism events present in patients with PVD, diabetes, obesity and CVD in comparison to those without any comorbidities.

**Table 5:** The impact of comorbidities on the cost of recovery and recovery time of the unilateral ankle fracture postoperative period.

Co‐morbidities				
	PVD	Diabetes	Obesity	CVD
**Cost without condition**	2521	2494	2426	2474
**Interquartile range**	(1323–5020)	(1311–5002)	(1299–4814)	(1309–4981)
**Cost with condition**	3057	3121	2826	3201
**Interquartile range**	(1506–7021)	(1445–6426)	(1436–5971)	(1615–6800)
**Cost comparison *p*‐value**	0.03	< 0.01	< 0.01	< 0.01
**PT days without condition**	85	84	82	85
**Interquartile range**	(36–476)	(35–460)	(35–437)	(35–469)
**PT days with condition**	170	144	112	149
**Interquartile range**	(49–665)	(44–642)	(41–581)	(49–654)
**PT days comparison *p*‐value**	< 0.01	< 0.01	< 0.01	< 0.01
**Number of patients without condition (*n*)**	6718	6287	5027	6438
**Number of patients with condition (*n*)**	394	825	2085	674
**Infection without event (%)**	2.8	2.7	2.6	2.6
**PE without event (%)**	3.2	3.1	2.7	2.9
**Infection with event (%)**	4.3	4.1	3.6	5.6
**PE with event (%)**	9.6	7.0	5.7	10.2

*Note:* Mood's Median tests were used for comparisons.

Costs and days in physical therapy (PT) are presented as medians and interquartile ranges.

Abbreviation: PE, pulmonary embolism.

## 4 Discussion

This large claims‐based analysis establishes a benchmark for the short‐term recovery period following unilateral ankle fracture and quantifies the clinical and economic burden of complication‐related events. Recovery duration and costs varied widely, with 38% of patients required more than 6 months to complete their post‐treatment care. These large variations in recovery timelines demonstrate the complexity of ankle fracture recovery.

A key finding of this study was that major post‐treatment events, including revision surgeries, MRS, complication surgeries and non‐operative hospitalizations are strongly associated with prolonged recovery and markedly higher healthcare costs. As this study is claims‐based, the relationships described represent associations rather than causal effect. The absence of clinical data such as fracture severity, fixation method, or functional outcome limits the ability to determine whether complications caused delayed recovery or whether patients predisposed to delayed recovery were more likely to require further interventions.

Direct comparison of recovery durations with prior clinical studies is limited by differences in study design, follow‐up length and outcomes reported. A previous study of operatively treated ankle fractures reported on delayed recovery in which 72% of patients still suffered from ankle stiffness and 52% had not returned to pre‐injury activity levels at 1 year after treatment, consistent with the prolonged rehabilitation found in this study [17]. The current study demonstrates that the need for MRS adds nearly 1 year to the recovery period and may be related to the incidence of infection. Major wound complications after ankle fracture are known to impair motion recovery and may also be related to infection [2, 8, 9]. One study showed that smoking, postoperative malreduction and hardware removal prior to fracture union were the most important factors predisposing to a permanent complication following an ankle fracture infection [18]. Post‐traumatic osteoarthritis occurs in roughly 25% of ankle fractures, with higher rates reported in fractures classified as Weber B+C+ [9]. In the present study, only five patients received salvage surgery at a rate of 0.07%. Previously, high complication rates (28%–35%) and reoperation rates matching or exceeding the 7% reported in this study (7%–13.6%) have been reported [19, 20, 21].

The presence of ankle or Achilles tendon contracture (M24.57 or M67.0) either before or after treatment for unilateral ankle fracture was associated with a substantial increase in both cost and recovery time. Patients who developed a contracture after surgery had approximately twice the total cost and 4 times the recovery duration compared to those without contracture. Similarly, preoperative motion loss related to contracture was linked to markedly prolonged recovery (88 vs. 537 days). Comorbidities also influenced recovery outcomes, with diabetes extending recovery by approximately 60 days and showing a higher rate of postoperative pulmonary embolism, though not infection, consistent with previous findings [22, 23]. In alignment with earlier literature, pulmonary embolism events were more frequent among patients with peripheral vascular disease, diabetes, obesity and cardiovascular disease, with only the latter showing a clinically meaningful increase in infection risk [23, 24, 25].

While prior studies have evaluated the national burden of ankle fractures using claims data [3, 26, 27], few have examined the recovery timeline or functional trajectory after treatment in both operative and non‐operative patients. Previous studies have assessed complication rates, reoperation frequency, and costs stratified by fracture severity. Although these studies provided valuable insights into the clinical and economic burden of ankle fractures, they did not address recovery duration or functional progress. In contrast, the current study defines a post‐treatment recovery benchmark for both operative and non‐operative patients by tracking physical therapy use, offering a more direct lens into patient rehabilitation. By focusing on the time from treatment to the final outpatient therapy session, our analysis highlights the substantial impact of complications such as joint contracture and motion‐restoring surgery on both cost and recovery time. Additionally, our study explores the additive burden of comorbidities and perioperative complications on short‐term outcomes. Together, these findings complement and expand on existing literature by shifting attention from fracture severity alone to the broader spectrum of factors that influence recovery after ankle fracture.

These data have important implications from a policy and resource allocation standpoint. Understanding the distribution of recovery durations and complication risks can inform bundled payment models and support evidence‐based planning for rehabilitation. Real‐world recovery benchmarks provide payers and health systems with realistic expectations for post‐fracture care including the identification of high‐cost pathways associated with MRS and rehospitalizations. An improved understanding of recovery pathways allows clinicians to develop nonoperative or preventative solutions for motion loss such as early, structured rehabilitation or use of stretch‐assist devices that may reduce the need for costly surgical procedures. Clearly, MRS is expensive and carries elevated risks of infection, pulmonary embolism and subsequent operations.

There are multiple limitations with a HCPCS claims‐based analysis. There is the presence of standard coding inconsistencies, particularly since this study spans the change from ICD 9 to ICD 10 codes. Additionally, the availability of patient demographic and clinical data is limited and clinical parameters such as fracture severity, radiographic outcomes, or functional outcomes are not captured. Recovery duration was defined by the last PT/OT claim, which may not perfectly correspond to the actual end of therapy. However, such limitations would be consistent across groups. Lastly, the database captures a large, commercially insured population which may not generalise to underinsured or publicly insured groups. Despite these limitations, the large sample size and standardised coding structure provide valuable real‐world recovery trends.

Finally, potential conflicts of interest must be considered. Some authors are affiliated with industry partners involved in the development of rehabilitation technologies. While this relationship presents the possibility of bias, all analyses were conducted using independent, de‐identified claims data, and the study's primary objective was descriptive rather than interventional. Transparency regarding these affiliations is essential for appropriate interpretation of the findings.

## 5 Conclusion

This study showed that short‐term recovery after ankle fracture is highly variable and strongly influenced by postoperative complications. These data establish a benchmark for recovery duration and cost, highlight opportunities for early intervention to prevent motion loss, and provide a foundation for future studies evaluating cost‐effectiveness and outcomes under value‐based care models.

## Author Contributions

Robert B. Weinstein, Samantha J. Beckley, Maha Karim, Shaun K. Stinton and Thomas P. Branch took part in study design, data analysis and interpretation, and manuscript preparation. All authors read and approved the final manuscript.

## Conflicts of Interest

The authors declare no conflicts of interest.

## Declaration of Financial/Other Relationships

TB is the founder and CEO of Ermi LLC. SB, MK and SS are paid employees of ArthroResearch LLC.

## Supporting information

Supporting File

## Data Availability

The data supporting this study's findings are sourced from IBM MarketScan and are subject to restrictions. These data were accessed under license for this study and are not publicly available.
