# Supplementary material for: Healthcare Utilization and Recovery Duration After Ankle Fracture: A Claims‐Based Study of Timeline, Costs and Complications
Source: J Eval Clin Pract. 2026 May 3;32(4):e70450. doi: 10.1111/jep.70450 (PMC13135818; doi:10.1111/jep.70450)
Supplement: Supplementary file 1 — Supporting File [file JEP-32-0-s001.docx]

Supplementary Materials

Table 1. Ankle Fracture Treatment CPT Codes


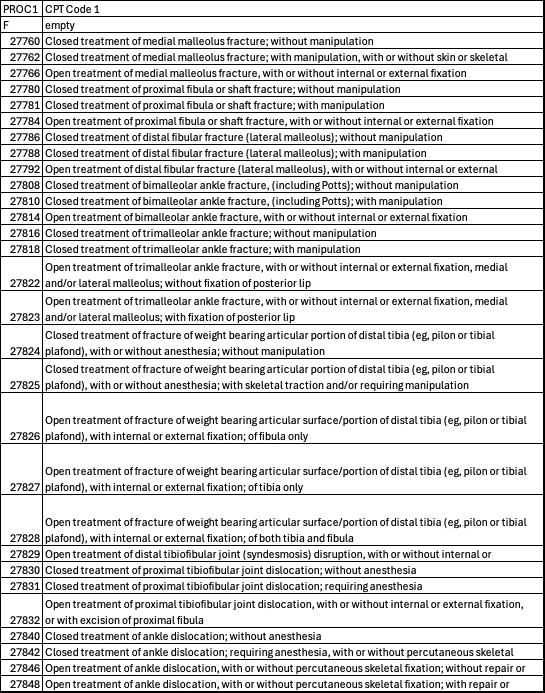


Table 2. Ankle Diagnostic Codes


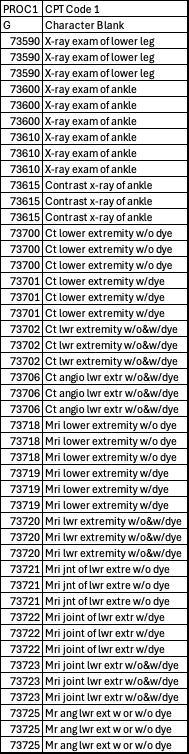


Table 3. Ankle Fracture Revision Codes


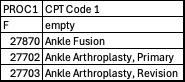


Table 4. Motion Restoring Surgery Codes


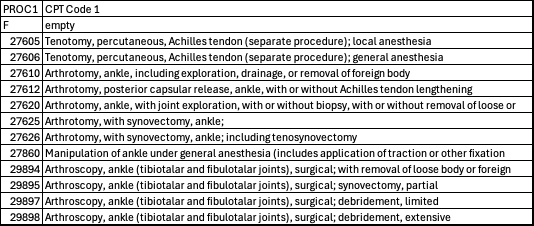


Table 5. Post-Ankle Fracture Complication Codes

All ICD-9 and ICD-10 codes containing “ankle” were used to identify ankle related hospitalizations.

Table 6. Ankle Joint Fibrosis (Achilles Contracture or Joint Contracture) ICD 9/10 Codes: all codes starting with

1. 718.47
2. M24.57
3. 727.81
4. M67.00

Table 7. Joint Infection ICD 9/10 Codes: all codes starting with

1. 711.
2. 996.
3. M00.
4. M01.
5. M02.

Table 8. Pulmonary Embolus ICD 9/10 Codes: all codes starting with

1. 415.1
2. 453.
3. I26.
4. I82.
